# Effectiveness of robot-assisted training added to conventional rehabilitation in patients with humeral fracture early after surgical treatment: protocol of a randomised, controlled, multicentre trial

**DOI:** 10.1186/s13063-017-2274-z

**Published:** 2017-12-06

**Authors:** Corinna Nerz, Lars Schwickert, Clemens Becker, Stefan Studier-Fischer, Janina Anna Müßig, Peter Augat

**Affiliations:** 1 Department of Clinical Gerontology, Robert-Bosch-Hospital, Auerbachstrasse 110, 70376 Stuttgart, Germany; 2 Department of Trauma and Orthopaedic Surgery, BG Trauma Centre Ludwigshafen, Ludwig-Guttmann-Strasse 13, 67071 Ludwigshafen, Germany; 3 Institute of Biomechanics, BG Trauma Centre Murnau, Professor-Küntscher-Strasse 8, 82418 Murnau am Staffelsee, Germany; 4 Institute of Biomechanics, Paracelsus Medical University, Strubergasse 21, 5020 Salzburg, Austria

**Keywords:** Robot-assisted rehabilitation, Humeral fracture, Randomised controlled study

## Abstract

**Background:**

The incidence of proximal humeral fractures increases with age. The functional recovery of the upper arm after such fractures is slow, and results are often disappointing. Treatment is associated with long immobilisation periods. Evidence-based exercise guidelines are missing. Loss of muscle mass as well as reduced range of motion and motor performance are common consequences. These losses could be partly counteracted by training interventions using robot-assisted arm support of the affected arm derived from neurorehabilitation. Thus, shorter immobilisation could be reached. Thus far, this approach has been tested in only a few small studies. The aim of the present study is to examine whether assistive robotic training augmenting conventional occupational and physical therapy can improve functional shoulder outcomes.

**Methods/design:**

Patients aged between 35 and 66 years with proximal humeral fracture and surgical treatment will be recruited at three different clinics in Germany and randomised into an intervention group and a control group. Participants will be assessed before randomisation and followed after completing an intervention period of 3 weeks and additionally after 3, 6 and 12 months. The baseline assessment will include cognition (Short Orientation-Memory-Concentration Test); level of pain in the affected arm; ability to work; gait speed (10-m walk); disability of the arm, shoulder and hand (Disabilities of the Arm, Shoulder and Hand Outcome Measure [DASH]); range of motion of the affected arm (goniometer measurement); visual acuity; and motor function of orthopaedic patients (Wolf Motor Function Test–Orthopaedic version [WMFT-O]). Clinical follow-up directly after the intervention will include assessment of disability of the arm, shoulder and hand (DASH) as well as range of motion and motor function (WMFT-O). The primary outcome parameter will be the DASH, and the secondary outcome parameter will be the WMFT-O. The long-term results will be assessed prospectively by postal follow-up. All patients will receive conventional occupational and physical therapy. The intervention group will receive additional robot-assisted training using the Armeo®Spring robot for 3 weeks.

**Discussion:**

This study protocol describes a phase II, randomised, controlled, single-blind, multicentre intervention study. The results will guide and possibly improve methods of rehabilitation after proximal humeral fracture.

**Trial registration:**

Clinicaltrials.gov, NCT03100201. Registered on 28 March 2017.

**Electronic supplementary material:**

The online version of this article (doi:10.1186/s13063-017-2274-z) contains supplementary material, which is available to authorized users.

## Background

The number of upper arm fractures is increasing in the aging workforce (52–66 years). The risk for proximal humeral fractures (PHFs) increases with age, starting at around the age of 45 years [1]. In 2008 approximately 370,000 emergency department (ED) visits occurred as a result of humeral fractures in the United States. Of these around 50% were due to PHF (184,300 ED visits), with double the number in females compared with males. In the age group 35–64 years, approximately 57,500 ED visits were recorded. About 275,000 ED visits due to PHF are predicted in 2030 in the United States. A similar trend in numbers is expected in Europe [1].

This relevant number in fractures of the upper extremity already places a high demand on surgical treatment and subsequent rehabilitation [2]. Currently, the existing physiotherapy and occupational therapy concepts are based on expert opinion. No robust evidence-based consensus exists for rehabilitation approaches after PHF [3, 4], which is somewhat surprising. Worldwide no randomised controlled trials (RCTs) have been published comparing rehabilitation protocols for PHFs. Thus, the optimal content, frequency, duration and intensity of rehabilitation after shoulder injuries remain unclear. Not surprisingly, authors of a recent systematic literature review found considerable variation and heterogeneity in rehabilitative treatment after PHF [4]. Researchers in several observational studies have criticized the inadequacy of currently available rehabilitation protocols for PHFs. The functional recovery of the fractured upper extremity was found to be disappointing in a German study [5]. One consistent problem is the long immobilisation of the affected arm of up to 6 weeks [6]. This leads to loss of muscle strength and results in loss of flexibility of the adjacent joints. A shorter time to recovery would be a prerequisite for an improved outcome after PHF [4].

In a pilot safety and feasibility study by our group, robot-assisted training using the Armeo®Spring device (Hocoma, Volketswil, Switzerland), which was originally designed for stroke rehabilitation [7], was shown to be safe and highly acceptable even among older patients. The pre-post measurements indicated a potential improvement in muscle strength, flexibility and motor control [8]. Assistive arm support was used to reduce partial loading of the affected arm and increase the movement request at an early stage after surgery. Thus, robot-assisted training could be a valuable option in orthopaedic fracture rehabilitation. However, evidence for effectiveness so far is confined mainly to the field of neurology, particularly in stroke patients. Literature on fracture rehabilitation using robot-assisted training is extremely scarce. Besides our pilot study, Padilla-Castenada et al. [9] evaluated the use of robot-assisted training after forearm and elbow fracture. They showed that the duration and number of repetitions of exercises can be increased by active assistive training.

Although preliminary, the promising findings of these pilot studies remain to be confirmed in an RCT. Hence, the aim of this study is to test whether additive robot-assisted training in patients with PHF using the Armeo®Spring device is superior to conventional occupational and physical therapy alone.

## Methods/design

### Study design

A single-blind, multicentre intervention RCT will be conducted. Robot-assisted training using the Armeo®Spring device added to conventional occupational and physical therapy will be compared with a control group receiving only conventional occupational and physical therapy.

### Randomisation

Participants will be randomised into one of two arms via block randomisation in each of the three centres, according to a predefined allocation sequence, after assessing inclusion and exclusion criteria. A randomisation list for each centre was created using the Internet-based Sealed Envelope software (https://sealedenvelope.com/; Sealed Envelope, London, UK). Assignment to the intervention and control groups will be performed by study nurses in each centre who will be blinded to interventions; unblinding will not be permissible. Anonymised data will be entered into a central database (Research Electronic Data Capture [REDCap], with data entry in agreement with the International Council for Harmonisation of Technical Requirements for Pharmaceuticals for Human Use standards for good clinical practice, see also SPIRIT procedure in supplementary material; Additional file 1: SPIRIT checklist and Additional file 2: Schedule of enrolment, interventions, and assessments) at the coordinating centre.

Participants will undergo clinical and quantitative assessments at one of the three sites: Robert-Bosch-Hospital Stuttgart, BG Trauma Centre Murnau or BG Trauma Centre Ludwigshafen. These three sites were chosen as comparators because they are the three largest clinical sites in Germany that treat PFH, do research or cooperate with an institute that conducts research, and possess Armeo®Spring devices. All procedures have been approved by the ethics committee of the University of Tübingen (381/2015BO1) and the regional medical association of Rheinland-Pfalz (837.519.15) and are in accord with the Declaration of Helsinki. Informed consent will be obtained from all participants (Additional file 3: Consent form). All participants will be insured. No data monitoring committee was consulted.

### Sample size

Adults in the working population aged between 35 and 66 years will be recruited at all three sites. In a pilot study [8], our group was able to measure an improvement in function with the Disabilities of the Arm, Shoulder and Hand Outcome Measure (DASH) score (median 47 [mean 46 ± 20 SD] to median 34 [mean 32 ± 15 SD]). As a result of a power analysis based on these results, 26 patients per group will be required to achieve 80% power at a significance level of α = 0.05. With an expected drop-out rate of 10%, a total of 60 patients should be recruited, comprising 20 patients at each of the 3 sites.

Inclusion criteria for both groups will be surgical treatment of the shoulder joint after upper arm fracture classified as AO Foundation/Association for the Study of Internal Fixation (Arbeitsgemeinschaft Osteosynthese; AO) type 11 [10]. Participants will be included between the fourth and seventh weeks after surgery. The participant population will be stratified by fracture location (subcapital/capital) and handedness.

Exclusion criteria will be determined during baseline assessment. Participants with limited cognition as defined by a score < 10 points on the Short Orientation-Memory-Concentration Test (SOMC), inadequate level of pain during movement of the affected shoulder joint (pain score > 5 on a pain visual analogue scale [VAS]), strongly limited vision or hearing, heart failure (New York Heart Association stage III-IV), chronic obstructive pulmonary disease with Global Initiative for Chronic Obstructive Lung Disease (GOLD) stage III-IV, walking speed < 0.8 m/second, isolated tuberculum majus fracture of the humerus (AO 11, A1), or fractures with involvement of the glenoid cavity, as well as double fractures or injury of the plexus or the axillary nerve, will be excluded. All participants will give their written informed consent to the study administrators at each centre prior to the clinical baseline assessment.

### Clinical assessment

**Fig. 1:**
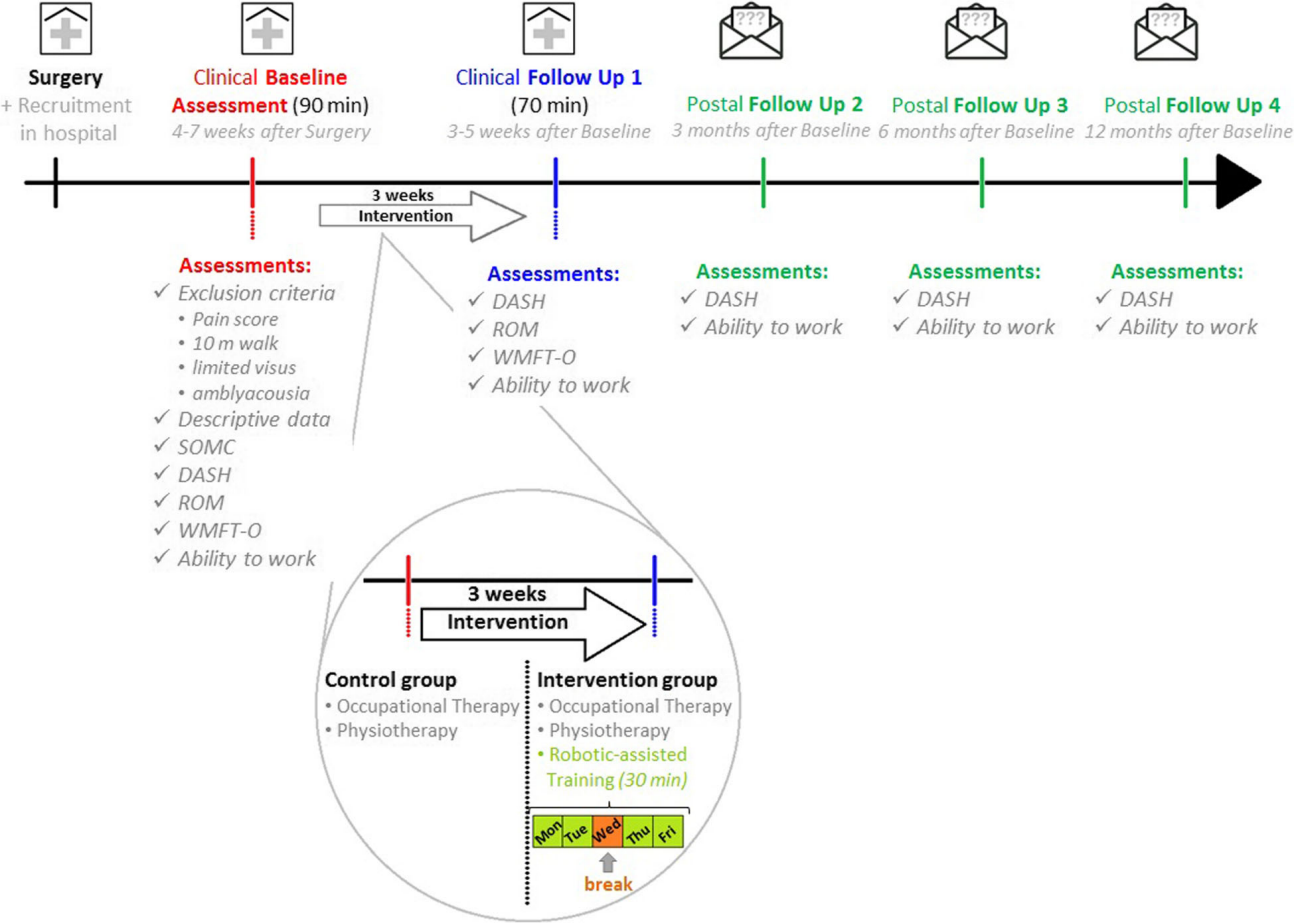
Plan of assessment and intervention procedures. *DASH* Disabilities of the Arm, Shoulder and Hand Outcome Measure, *ROM* Range of motion, *WMFT-O* Wolf Motor Function Test–Orthopaedic version

The estimated total duration of clinical baseline and follow-up assessments will be 160–180 minutes. The clinical assessment will be carried out for all participants before the first potential training session (baseline) and after the last potential training session (follow-up 1 at 3–5 weeks after baseline). For analysis of sustainability, further postal follow-up assessments (2, 3 and 4) will be carried out 3, 6 and 12 months after the baseline assessment (Fig. 1). In these follow-up assessments, only the DASH and the ability to work (what job to what extent) will be evaluated. All assessments will be performed by trained assessors.

Clinical data such as sex, age, body mass index, handedness, date and type of fracture, and date of surgical treatment will be recorded. Furthermore, the participant’s occupation before and after the accident as well as the number of days between the accident and the return to work will be recorded. If the participant will not be able to return to work after the accident, the reason for being incapacitated will be assessed.

The SOMC is an assessment of cognitive impairment and will be applied in this study. It can differentiate between mild, moderate and severe cognitive deficits. The SOMC was derived from the longer Blessed scale [11] to enable it to be performed by a non-physician. The SOMC score highly correlates (*r* = 0.92) with the full scale and is nearly as sensitive as the longer version. Any error score of 0–6 is within normal cognitive limits [12].

In this study the potential pain score in the shoulder during motion will be assessed by the patient and then documented by the therapist as a numerical value. Therefore, a VAS will be used, which was proven to be a valid method for determining pain in clinical routine [13]. As an assessment of overall motor function, the comfortable gait speed over a distance of 10 m will be assessed. The walking course will consist of a total of 14 m, 2 m for acceleration, 10 m for the speed measurement and 2 m for slowing down and stopping. The participants will be directed to walk at their own comfortable pace [14].

The flexibility (range of motion [ROM]) of the shoulder is measured using a goniometer according to the AO neutral-0 method in pain-free range. The maximum angle in elevation, retroversion, abduction and adduction as well as external rotation will be measured. The maximal grip strength will be assessed three times for both hands using a dynamometer (JAMAR Technologies/MSD, Londerzeel, Belgium) according to the American Society of Hand Therapists [15] guideline. The average of three measurements will be calculated for both hands.

In order to describe the extent to which the participants correctly follow medical advice and the therapy sessions, their adherence will be documented. This will be provided by recording the duration and frequency of additional training that contains conventional occupational and physical therapy (group or individualised) during the intervention.

#### Primary outcome

The primary outcome of self-perceived limitation of shoulder, arm and hand function will be assessed with the first module of the DASH [16], which is considered a valid and reliable test in clinical practice [17]. Using a total of 30 questions, we will assess the restriction relating to the function and activity of the shoulder, arm and hand in daily living, as well as self-esteem and potentially existing symptoms of the shoulder, arm and hand, such as pain or prickle. The endpoint will be a score calculated from the ratings of the individual responses (1 is the best value, 5 the worst).

#### Secondary outcome

For objective proof of a functional improvement between the beginning and the end of the intervention, the Wolf Motor Function Test–Orthopaedic version (WMFT-O) [18] was conducted. The WMFT-O is an adapted version of the basic WMFT [19] with good inter- and intra-rater reliability. The WMFT-O includes 20 arm motion tasks of daily living that will be examined and evaluated in terms of time, functional capacity and quality of movement, arranged hierarchically and increasing from coarse movements in the elbow and shoulder area to more complex and dexterous tasks in the fingers and the hand area.

### Intervention

Both the intervention group and the control group received physical and occupational therapy for a period of 3 weeks; concomitant interventions were not restricted. The additional robot-assisted therapy commenced as early as 4 weeks after surgery and not later than 7 weeks after surgery. There are no evidence-based guidelines or requirements on the content, duration or intensity of rehabilitation after shoulder injuries. Typically the therapeutic content consists of exercises for stabilisation and mobilisation of the shoulder and the surrounding joints, as well as muscle strengthening (three to six times per week for 25–30 minutes). The obtained conventional physical and occupational therapy will be documented but not influenced. The frequency and duration of individual training schedules, as well as adverse events, will be recorded in a protocol. Serious adverse events will be treated as criteria for discontinuing the interventions.

The intervention group received robot-assisted training in addition to the protocol intervention. The robot-assisted training will be performed using the clinically evaluated Armeo®Spring medical device. By means of sensory orthosis, arm movements will be supported and transmitted to a computer system and visually shown on a screen. Furthermore, audio feedback elements and augmented performance feedback will be used for guidance. The Armeo®Spring device encourages patients with motivating game-like exercises in an inspiring virtual environment. The sensor-based computer system calculates game kinetics for 3D animation of arm functions, including grasping and goal-orientated functional reaching, as well as object manipulation (e.g., throwing a Frisbee). The mechanism to support the weight of the arm will be increased or decreased gradually to adapt to the ability of the user. Thus, the participant will have the ability to perform targeted and controlled movements in accordance with medical safety standards, including the movement limit as suggested by the operating physician.

Preceding every therapy unit, saddle height and joint positioning of the functional segment parts of the exoskeleton will be individually adjusted to the upper extremity proportion of the affected arm of the participant. With the participant in an upright seating position with the spine on the back rest, the participant’s arm will be attached to the exoskeleton with Velcro belts. A calibration of the ROM using the orthosis will be performed by the participants and assessed to calculate an individual game-specific motion envelope. This calibration will help to configure a safe environment for shoulder movements of the participants and will prevent overload and excessive demands. All participants of the intervention group at all three sites (*n* = 30) will receive a 3-week robotic intervention in a standardised training setting with supervised exercise four times per week for 30 minutes per session.

**Fig. 2:**
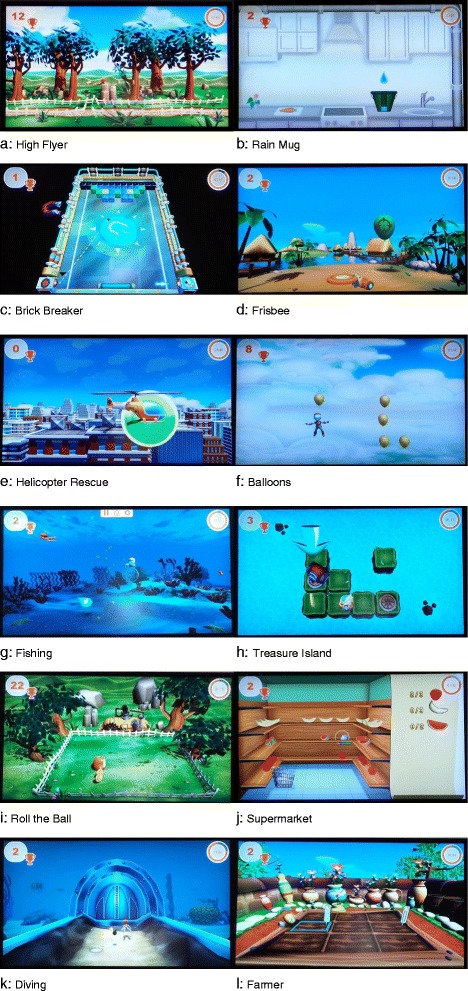
**a**–**l** Armeo®Spring games

The normative levels of exercise capacity are inspired by a previous pilot study [8], and a rough guideline was created in consensus with the three centres. To create this guideline the 17 existing exercises were categorised according to the direction of the movement. Then 12 of the 17 existing exercises were selected for training of patients after PHF on the basis of the categorised movement directions. These 12 exercises are illustrated and described in Fig. 2 and Table 1.

**Table 1:** Description of the 12 exercises

Name of the task	Task description	Functional challenge
A. High flyer	Collect the coins while flying. Avoid obstacles like bombs and birds.	Flex and extend your shoulder to move up and down.
B. Rain mug	Catch the raindrops with your mug.	Abduct and adduct your shoulder horizontally to move the mug to the left and right sides.
C. Brick breaker	Destroy the bricks with the ball by controlling the moving platform. Making sure not to lose the ball.	Abduct and adduct your shoulder horizontally to move to the left and right sides.
D. Frisbee	Target the Frisbee at the balloons and bring the balloons to burst.	Flex, extend, adduct and abduct your shoulder. Bend and straighten your wrist to burst the balloons.
E. Helicopter rescue	Steer helicopters and rescue your robot friends off the roofs of buildings. Hover over them in order to pick them up.	Abduct and adduct your shoulder horizontally to move to the left and right sides. Rotate the forearm inward and outward to open and close the loading door of the helicopter.
F. Balloons	Catch the balloons while avoiding obstacles like bombs and birds.	Flex and extend your shoulder to move up and down. Abduct and adduct your shoulder horizontally to move to the left and right sides.
G. Fishing	Collect the fish and other aquatic animals, but no waste.	Flex and extend your shoulder to move up and down. Abduct and adduct your shoulder horizontally to move to the left and right sides.
H. Treasure Island	Take the key and roll the ball along the path over the bridge up to the treasure chest. Try to collect coins while doing this, and avoid the obstacles.	Flex and extend your shoulder to move up and down. Abduct and adduct your shoulder horizontally to move to the left and right sides.
I. Roll the ball	Move the ball and collect coins, avoiding the obstacles, like bugs and bombs.	Flex, extend, adduct and abduct your shoulder to move the ball.
J. Supermarket	Take the products, which are on the shopping list, from the shelves and put them in the trolley.	Flex, extend, adduct and abduct your shoulder to move the hand with which you can grab the products.
K. Diving	Collect all the coins in the underwater world while avoiding the bombs.	Flex, extend, adduct and abduct your shoulder to move the diver.
L. Farmer	Maintain your garden by using a variety of transaction types, from sowing to harvesting.	Flex, extend, adduct and abduct your shoulder to move the hand with which you can (e.g., watering the seed).

**Fig. 3:**
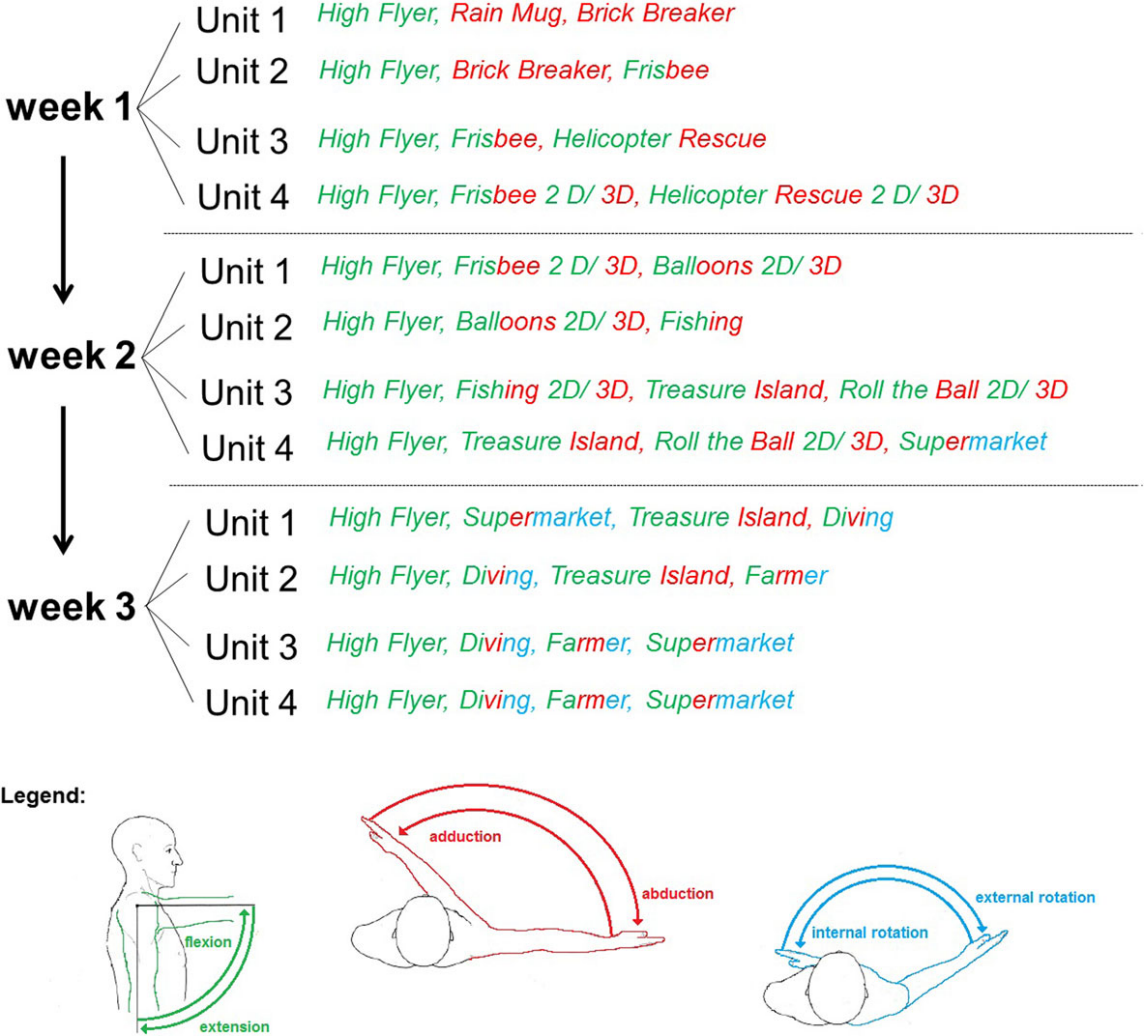
Training schedule with games and directions of movement

A plan for the 3-week intervention was created in which the exercises gradually increased from 1D up to 3D movements. Each exercise will take 1–3 min, depending on the task and level of difficulty chosen. Each training unit will start with exercise in a pre-defined order, as shown in Fig. 3, whereas progression will depend on the participant’s current physical performance ability, which will be decided by the therapist. Progression within one unit will be achieved by gradually increasing the level of difficulty and the number of repetitions of a single exercise and thus the duration of the exercise within one training unit. Progression of shoulder function over the intervention period will be achieved by gradually increasing dimensionality of the exercise and by performing different exercises, which require the participant to use more joint segments.

### Statistics

The centre and the number of treatment units will be regarded as possible covariates for the primary outcome. All data assessed will be described descriptively (mean, SD, 95% CI and median for metric variables; frequency and percentile for categorical variables). After checking the data for normal distribution by comparing the distribution histograms and Shapiro-Wilk test, the group comparison will be carried out with the *t* test or the rank-based Wilcoxon test. For the primary outcome analysis of covariance with study centre, number of therapy units (to take account of the training inhomogeneity) and injury severity (to take account of the patient’s homogeneity) as covariates will be performed. The secondary outcome (WMFT) will be analysed to generate study hypotheses using the methods described above. Inferential statistics will follow an intention-to-treat approach with mean imputation or regression imputation for all randomised patients with incomplete datasets if deemed necessary. Per-protocol analysis will be performed for evaluation of the efficacy of the intervention in patients with complete datasets and without protocol violations.

## Discussion

PHFs are often associated with immobilisation of the affected arm, leading to functional loss, pain and psychosocial problems. The aim of rehabilitation is to reintegrate the patient as quickly as possible into daily routine and work after surgical treatment. This study protocol describes an experimental set-up allowing the analysis of an additive robot-assisted training in combination with conventional occupational and physical therapy in comparison with conventional occupational and physical therapy alone. The robot-assisted training will allow the patient to mobilise the affected arm earlier and thereby to lose less muscle mass and improve motor capacity. This will be measured objectively with the WMFT-O and subjectively with the self-completed DASH questionnaire. This will possibly allow for an earlier return to work.

To the best of our knowledge, this is the first controlled study in the field of rehabilitation of PHFs using robot-assisted training. The results of the study will give insight into treatment modalities after PHF and into restoration of shoulder function. This will be important for learning about ways to improve therapy in terms of duration, intensity, frequency and extent. New ways will be needed also to improve commitment to therapy. Therefore, the robot-assisted approach will be an option to offer more effective rehabilitation early after surgery.

### Limitations

The sample size is relatively small and powered only for a functional endpoint (DASH). Social or economic endpoints such as return to work or cost-effectiveness of this intervention would require larger sample sizes and were not the purpose of this early-stage RCT. Furthermore, the set-up with three different associated centres will lead to some difficulties in the standardisation of the assessments. It is expected that different observers and therapists in the three institutions have subjective differences in the accomplishment of the tests. Assessment of the WMFT will be videotaped to standardise this as much as possible.

Heterogeneous treatment modalities will also exist to some extent for rehabilitation after PHF in the conventional occupational and physical therapy in terms of duration, intensity and frequency. Theoretically, participation in the robotic group could lead to increased or decreased motivation to participate in other types of exercises. Adherence will therefore be documented.

For dissemination the full and optimised training protocol will be provided via an online research platform. Also two videos, one about the instructions of the WMFT-O and one about the rating, are provided on YouTube (https://www.youtube.com/watch?v=8yDcWK9Xrtw, https://www.youtube.com/watch?v=MsfrGauYHfc).

## Conclusions

The results of the present RCT might help researchers in designing new therapeutic interventions in the rehabilitation of humeral fractures.

### Trial status

Patient recruitment was at 50% at the time of publication of this article.

## Additional files

Additional file 1: Standard Protocol Items: Recommendations for Interventional Trials (SPIRIT) checklist. (DOC 121 kb)

Additional file 2: Schedule of enrolment, interventions, and assessments. (PDF 25 kb)

Additional file 3: Consent form. (DOCX 94 kb)

## Abbreviations

AO: AO Foundation/Association for the Study of Internal Fixation (Arbeitsgemeinschaft Osteosynthese)
DASH: Disabilities of the Arm, Shoulder and Hand Outcome Measure
ED: Emergency department
GOLD: Global Initiative for Chronic Obstructive Lung Disease
PHF: Proximal humeral fracture
RCT: Randomised controlled trial
REDCap: Research Electronic Data Capture
ROM: Range of motion
SOMC: Short Orientation-Memory-Concentration Test
VAS: Visual analogue scale
WMFT: Wolf Motor Function Test
WMFT-O: Wolf Motor Function Test–Orthopaedic version
