# Supplementary material for: Effectiveness of robot-assisted training added to conventional rehabilitation in patients with humeral fracture early after surgical treatment: protocol of a randomised, controlled, multicentre trial
Source: Trials. 2017 Dec 6;18:589. doi: 10.1186/s13063-017-2274-z (PMC5719790; doi:10.1186/s13063-017-2274-z)
Supplement: Supplementary file 2 — Schedule of enrolment, interventions, and assessments. (PDF 25 kb) [file 13063_2017_2274_MOESM2_ESM.pdf]

Figure 4. **S**chedule of enrolment, interventions, and assessments

		STUDY PERIOD					
				Post-allocation			
	**Enrolment (surgery)**	***Baseline (clinical)***	**Allocation**	***Follow up 1 (clinical)*** *3-5 weeks*	***Follow up 2 (postal)*** *3 months*	***Follow up 3 (postal)*** *6 months*	***Follow up 4 (postal)*** *12 months*
**TIMEPOINT**	$-t_1$	0	t_1	t_2	t_3	t_4	t_5
**ENROLMENT:**							
**Eligibility screen**	X						
**Informed consent**	X						
**Randomisation**			X				
**3 WEEKS INTERVENTION:**							
[Control group]			◆———	———◆			
[Robotic-assisted training group]			◆———	———◆			
**ASSESSMENTS:**							
[DASH[1] first outcome]		X		X	X	X	X
[WMFT-O[2] second outcome]		X		X			
[ROM[3]]		X		X			
[Ability to work]		X		X	X	X	X
[SOMC[4]]		X					
[descriptive data[5]]		X					

[1] Disability of the Arm, Shoulder and Hand questionnaire; [2] Wolf-Motor-Function-Test-Orthopaedics; [3] Range of Motion; [4] Short-Orientation-Memory-Concentration Test; [5] sex, age, body mass index, handedness, date and type of fracture and date of surgical treatment
