# Supplementary material for: Effectiveness of robot-assisted training added to conventional rehabilitation in patients with humeral fracture early after surgical treatment: protocol of a randomised, controlled, multicentre trial
Source: Trials. 2017 Dec 6;18:589. doi: 10.1186/s13063-017-2274-z (PMC5719790; doi:10.1186/s13063-017-2274-z)

Appendix

1: Consent form


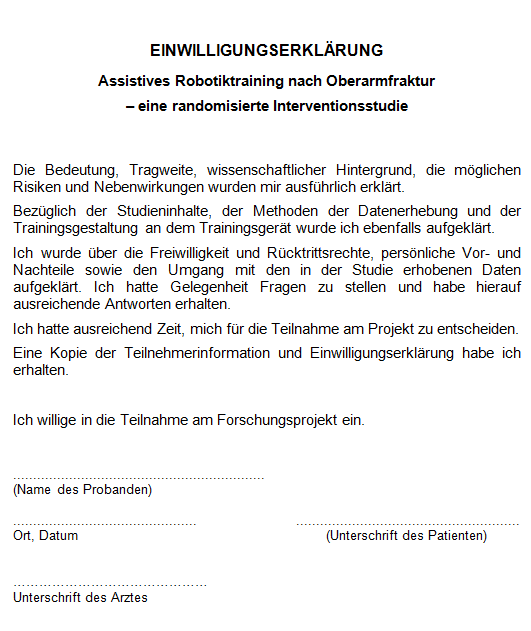


2: [data](https://dict.leo.org/englisch-deutsch/data) [privacy](https://dict.leo.org/englisch-deutsch/privacy) [statement](https://dict.leo.org/englisch-deutsch/statement)


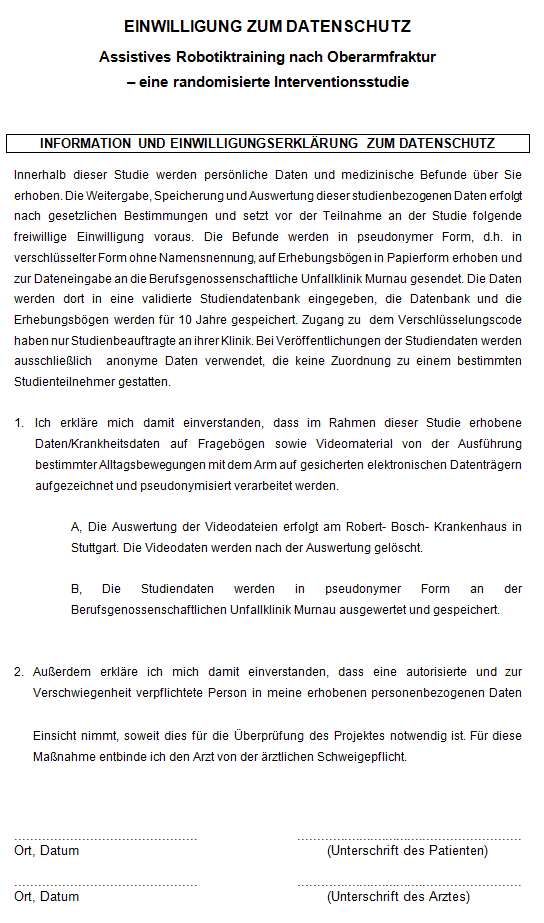

Supplement: Supplementary file 3 — Consent form. (DOCX 94 kb) [file 13063_2017_2274_MOESM3_ESM.docx]
